# Variation in Prostate-Specific Antigen Testing Rates and Prostate Cancer Treatments and Outcomes in a National 20-Year Cohort

**DOI:** 10.1001/jamanetworkopen.2021.9444

**Published:** 2021-05-17

**Authors:** Oskar Bergengren, Marcus Westerberg, Lars Holmberg, Pär Stattin, Anna Bill-Axelson, Hans Garmo

**Affiliations:** 1 Department of Surgical Sciences, Uppsala University, Uppsala, Sweden; 2 Department of Mathematics, Uppsala University, Uppsala, Sweden; 3 Translational Oncology & Urology Research (TOUR), School of Cancer and Pharmaceutical Sciences, King’s College London, London, United Kingdom; 4 Regional Cancer Center Uppsala Örebro, Uppsala, Sweden

## Abstract

**Importance:**

The diagnostic activity for prostate cancer has increased during the past decades. However, the benefit and harm of the increased diagnostic activity have not been quantified in detail for a country or a large region.

**Objective:**

The aim of this study was to evaluate and quantify the association between increases in diagnostic activity driven by prostate-specific antigen testing and incidence of prostate cancer diagnosis, treatment, and mortality.

**Design, Setting, and Participants:**

This cohort study used the Proxy-Based Risk-Stratified Incidence Simulation Model–Prostate Cancer to examine observed data on all Swedish men with prevalent prostate cancer and compare them with a corresponding, hypothetical, simulated scenario with more restrictive diagnostic activity. All men aged 40 to 100 years living in Sweden during the time period 1996 to 2016 with incident and prevalent prostate cancer were included. The second scenario is the corresponding, hypothetical, simulated scenario where diagnostic activity remained constant as of 1996 (the beginning of the prostate-specific antigen testing era) throughout the study period.

**Exposures:**

High or low diagnostic activity for prostate cancer.

**Main Outcomes and Measures:**

Incidence of prostate cancer diagnosis, treatment (deferred treatment, curative treatment, and hormonal treatment), and prostate cancer mortality.

**Results:**

During the study period from 1996 to 2016, 188 884 men were diagnosed with prostate cancer at a median (interquartile range) age of 71 (64-77) years. Compared with the low–diagnostic activity scenario, in the high–diagnostic activity scenario, the number of men diagnosed with prostate cancer was 48% higher (423 vs 286 [95% CI, 271-302] per 100 000 men per year), 148% more men were diagnosed with low- or intermediate-risk cancer (221 vs 89 [95% CI, 73-105] per 100 000 men per year), and 108% more men received curative treatment (152 vs 73 [95% CI: 66-85] per 100 000 men per year). There were up to 15% fewer prostate cancer deaths in the scenario with high–diagnostic activity (incidence rate ratio, 0.85; 95% CI, 0.82-0.88).

**Conclusions and Relevance:**

This study’s results suggest that increased prostate-specific antigen testing and diagnostic activity are associated with a larger number of men being diagnosed with prostate cancer, predominately with low- and intermediate-risk disease. The increased diagnostic activity was associated with a 2-fold increase in curative treatment and a modest decrease in mortality.

## Key Points

**Question:**

Has the increased diagnostic activity driven by prostate-specific antigen testing been associated with more treatment for prostate cancer and decreased mortality?

**Findings:**

In this population-based cohort study, Swedish men with prostate cancer from 1996 to 2016 were compared with a corresponding, simulated cohort with more restrictive diagnostic activity. The observed prostate-specific antigen diagnostic activity was associated with up to 15% fewer deaths, a 48% increase in prostate cancer diagnosis, and 108% more men receiving curative treatment compared with the simulated cohort.

**Meaning:**

The opportunistic, high diagnostic activity for prostate cancer was associated with a 2-fold increase in curative treatment with a modest decrease in mortality.

## Teaser

This cohort study uses a simulation model to assess the association between increases in screening for prostate cancer and prostate cancer diagnosis, treatment, and mortality.

## Introduction

The diagnostic activity for prostate cancer has steadily increased during the past 30 years, largely due to increased use of prostate-specific antigen (PSA) testing. This led to an increase in the detection of low and intermediate-risk disease and an ensuing use of curative procedures with a high frequency of adverse effects.^1^ The intensified diagnostic testing was introduced without clear guidelines or a systematic evaluation. The benefits and harms of the increased diagnostic activity have not been quantified in detail for a country or a large region.

Previous studies on opportunistic diagnostic activity for prostate cancer have mainly compared incidence before and after the introduction of PSA testing and have shown large increases in the incidence of prostate cancer, predominately localized disease representing a substantial overdiagnosis.^2,3,4,5,6^

The aim of this study was to quantify the outcomes associated with increasing diagnostic activity during the past couple decades since PSA testing began, comparing it with a hypothetical scenario with more restrictive diagnostic activity to see whether the increased diagnostic activity was associated with more treatment and decreased mortality by use of a new model that simulates incidence, treatment trajectories, and mortality when given a specific scenario of diagnostic activity.^7^

## Methods

### Data Sources

This cohort study was approved by the Research Ethics Board at Umea University. Informed consent was not required because the study used preexisting, deidentified data, in accordance with 45 CFR §46. This study follows the Strengthening the Reporting of Observational Studies in Epidemiology (STROBE) reporting guideline.

Data were retrieved from the National Prostate Cancer Register of Sweden (NPCR), a comprehensive database of Swedish men with prevalent prostate cancer during the period of 1992 to 2016. Information on men in NPCR together with a random sample of prostate cancer–free men have been linked to several other registers in Prostate Cancer Data Base Sweden (PCBaSe, version 4.0).^8^ Population demographic data from Statistics Sweden together with additional information from NPCR, the Swedish Cancer registry, the Prescribed Drug Registry, the Patient Registry, and the Cause of Death Registry were retrieved to estimate model parameters.^7,8,9^ Risk categories (low, intermediate, high, locally advanced, and distant metastasized) were defined using a modification of the National Comprehensive Cancer Network risk categorization.^9^

### Simulation Model

The simulation model, Proxy-Based Risk-Stratified Incidence Simulation Model–Prostate Cancer (PRISM-PC), which has been previously described^7^ and is summarized in the eAppendix in the Supplement, is based on 25 years of data from PCBaSe. In short, the model works by first simulating incidence and mortality for a population of men aged 40 to 100 years during a specified time period in steps of 1 year. In each step, a man without prior diagnosis is either diagnosed with prostate cancer, stays alive without diagnosis, or dies of other causes, and each man with a prior diagnosis either stays alive or dies from prostate cancer or from other causes. Because we had no longitudinal data on PSA or biopsies available, the model is based on a proxy for the diagnostic activity. The proxy is defined at each calendar year for each cohort (defined by year of birth and region of residence) as the incidence of a low- or intermediate-risk prostate cancer by numbers at risk.

We extended the model to include treatment trajectories of each simulated man with a prostate cancer diagnosis. These were simulated using a resampling technique where the model sampled a treatment trajectory from a similar observed man with the same age at diagnosis, risk category, and time and cause of death. Treatments for prostate cancer were divided into deferred treatment, including watchful waiting and active surveillance, radical prostatectomy, radical radiotherapy, and androgen deprivation therapy. Data on treatment trajectories were obtained in PCBaSe^8^ for each man diagnosed with prostate cancer between 1996 and 2016 (eAppendix in Supplement).

### Study Design

We used the extended simulation model to compare 2 scenarios. The first scenario, high diagnostic activity, is the observed clinical practice between 1996 and 2016. The second scenario, low diagnostic activity, was a simulated, hypothetical situation where the diagnostic activity remained constant as of 1996 (ie, the beginning of the PSA testing era) throughout the study period. As there are both lead time effects on survival and changes in treatment strategies from 1996 to 2016 present in the data, the true survival for men with prostate cancer in the scenario with low diagnostic activity was unknown. Therefore, we performed 2 different simulations of this scenario based on 2 extreme assumptions on survival from diagnosis: an optimistic extreme, in which we assumed that men diagnosed in the low–diagnostic activity scenario had similar lead time and treatment effects as corresponding men in the high–diagnostic activity scenario (eg, a simulated man diagnosed in 2008 would have the same lead time and benefit from improved treatments as a real man diagnosed in 2008), and a pessimistic extreme, where men neither benefitted from lead time nor treatment effects (eg, a simulated man diagnosed in 2008 would have the same lead time and treatment effects as a real man diagnosed in 1996). None of these scenarios are accurate; however, the real survival estimate must be somewhere in between assuming that early diagnosis of prostate cancer is beneficial and prostate cancer care improved over time.

### Statistical Analysis

For each scenario, we ran the entire simulation 100 times (as described by Westerberg et al^7^) and calculated the number of men diagnosed, year of diagnosis, risk category, person-years in each treatment (from start of treatment to end of follow-up) and prostate cancer–specific mortality. Incidence, treatment prevalence, and mortality are age-standardized (age distribution of men was 40 to 100 years in 2016), calculated yearly per 100 000 men at risk, and presented as the mean per year over the entire study period, 1996 to 2016. Confidence intervals were constructed accounting for parameter uncertainty and simulation variability.^7^ The incidence rate ratio (IRR) of prostate cancer mortality was computed comparing the incidence rate in the high–diagnostic activity scenario with each of the extreme low–diagnostic activity scenarios. Statistical analysis was performed using R software version 4.0.2 (R Project for Statistical Computing) from February 2020 to January 2021.

## Results

### Study Population

During the study period from 1996 to 2016, 188 884 men were diagnosed with prostate cancer at a median (IQR) age of 71 (64-77) years. The median number of men aged 40 to 100 years living in Sweden was 2.23 million during the study period, ranging from 2.03 million in 1996 to 2.49 million in 2016. The median (IQR) age in 1996 was 56 (48-68) years and increased to 58 (50-70) years in 2016.

### Prostate Cancer Cases and Risk Category

The number of men diagnosed with prostate cancer was 48% higher in the scenario with high diagnostic activity compared with the scenario with low diagnostic activity (423 vs 286 [95% CI, 271-302] per 100 000 men per year).

**Figure 1.**
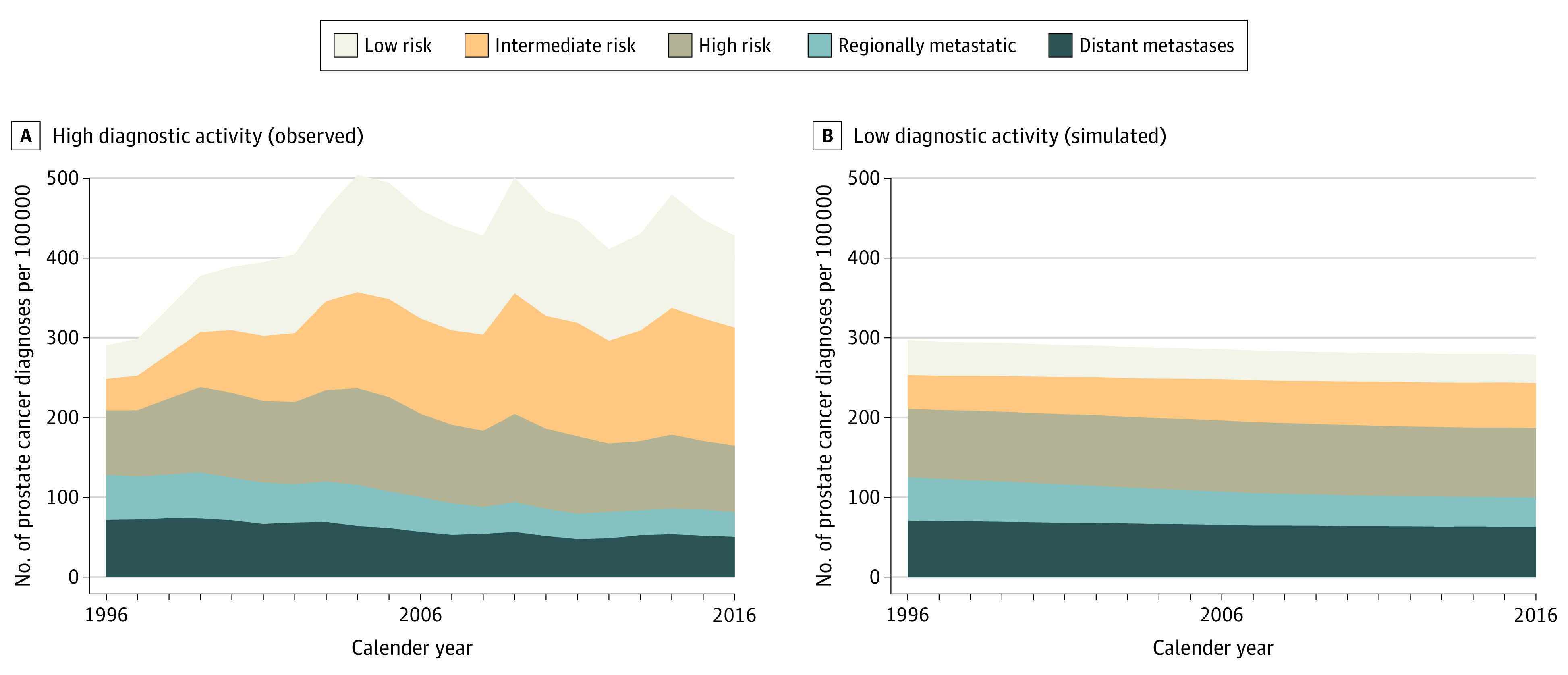
Incidence of Prostate Cancer by Risk Category Proxy-Based Risk-Stratified Incidence Simulation Model–Prostate Cancer (PRISM-PC). Per 100 000 men, age-standardized (age distribution of men 40 to 100 years in 2016).

Regarding risk category, 148% more men were diagnosed with low or intermediate-risk disease in the scenario with high diagnostic activity (221 vs 89 [95% CI, 73-105] per 100 000 men per year). Differences between the 2 scenarios regarding locally advanced or distant metastasized disease (103 vs 110 [95% CI, 96-123] per 100 000 men per year) (Figure 1) were not statistically significant.

### Treatment

**Figure 2.**
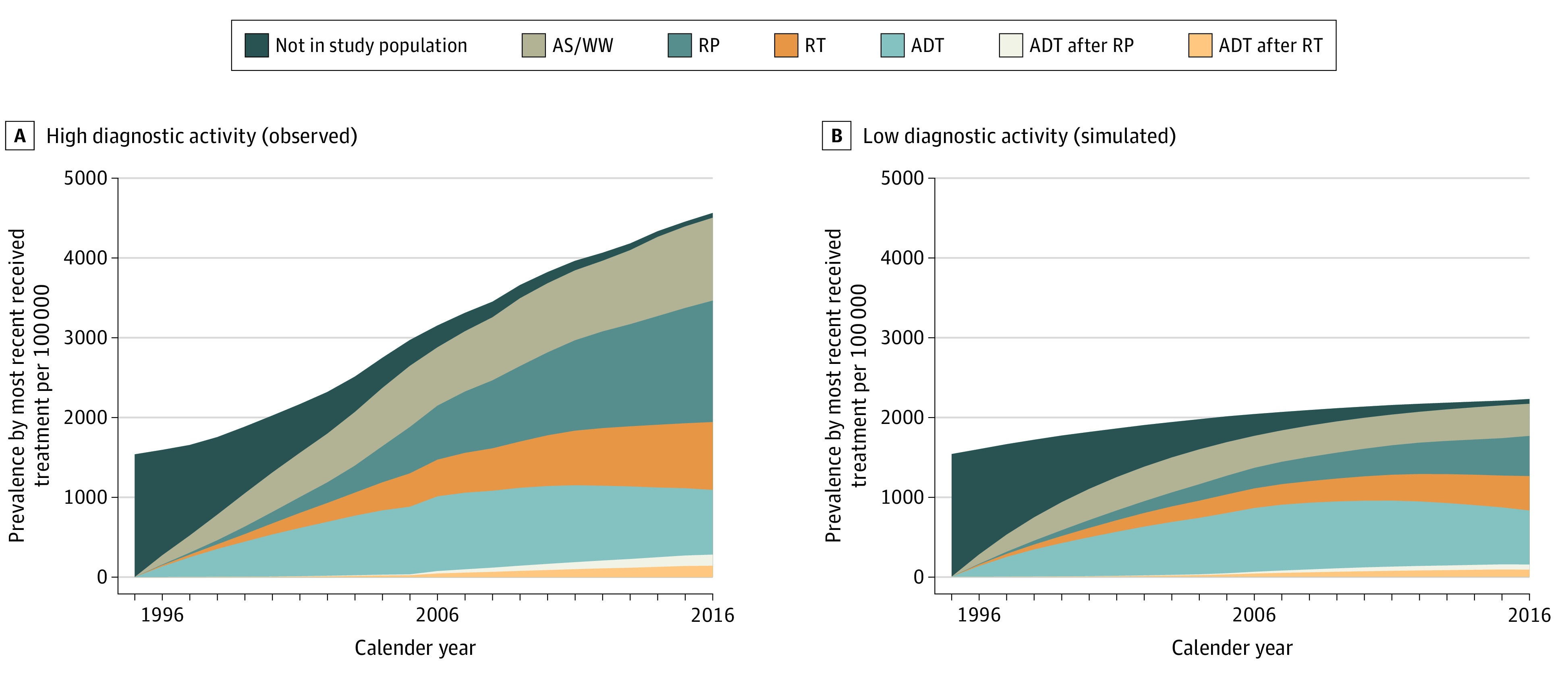
Treatment Prevalence for Prostate Cancer Per 100 000 men, age-standardized (age distribution of men 40 to 100 years in 2016). ADT denotes androgen deprivation therapy; AS/WW, active surveillance or watchful waiting; RP, radical prostatectomy; RT, radiotherapy.

In the scenario with high diagnostic activity, 78% more men received deferred treatment (132 vs 74 [95% CI, 68-80] per 100 000 men per year) and 108% more men received curative treatment (152 vs 73 [95% CI, 66-85] per 100 000 men per year) compared with the scenario with low diagnostic activity. The number of men who received hormonal treatment was very similar between the high- and low-activity scenarios (140 vs 139 [95% CI, 126-148] per 100 000 men per year) (Table, Figure 2).

**Table.** Patient and Prostate Cancer Tumor Characteristics

Characteristic	Patients, No. (%) [95% CI]			
	Observed	Observed per 100 000	Simulated	Simulated per 100 000
No.	188 884 (100.0)	8883 (100.0)	128 701 (100.0) [123 524-133 878]	6014 (100.0) [5693-6334]
Age at diagnosis, y				
<60	20 261 (10.7)	847 (9.5)	8130 (6.3) [7013-9247]	340 (5.7) [272-409]
60-69	66 342 (35.1)	3054 (34.4)	34 004 (26.4) [30 706-37 302]	1591 (26.5) [1410-1772]
70-79	67 316 (35.6)	3468 (39)	53 182 (41.3) [49 758-56 606]	2655 (44.1) [2437-2873]
≥80	34 965 (18.5)	1515 (17)	33 385 (25.9) [31 794-34 976]	1428 (23.7) [1306-1550]
Year of diagnosis				
1996-2000	33 267 (17.6)	1692 (19.1)	29 151 (22.7) [28 406-29 896]	1473 (24.5) [1362-1584]
2001-2005	45 198 (23.9)	2258 (25.4)	29 350 (22.8) [28 418-30 282]	1444 (24) [1331-1557]
2006-2010	48 817 (25.8)	2289 (25.8)	30 471 (23.7) [28 816-32 127]	1417 (23.6) [1286-1547]
2011-2016	61 602 (32.6)	2643 (29.8)	39 729 (30.9) [36 793-42 664]	1680 (27.9) [1511-1849]
Stage group				
Low	49 857 (26.4)	2312 (26)	17 216 (13.4) [14 869-19 563]	807 (13.4) [678-937]
Intermediate	49 876 (26.4)	2330 (26.2)	22 752 (17.7) [18 664-26 840]	1063 (17.7) [854-1271]
High	43 476 (23)	2071 (23.3)	39 448 (30.7) [35 935-42 962]	1844 (30.7) [1648-2040]
Locally advanced	18942 (10)	905 (10.2)	19 426 (15.1) [16 836-22 017]	909 (15.1) [763-1056]
Metastasized	26 733 (14.2)	1265 (14.2)	29 858 (23.2) [27 569-32 147]	1390 (23.1) [1243-1537]
PSA				
0-9	86 662 (45.9)	4007 (45.1)	38 970 (30.3) [36 397-41 544]	1805 (30) [1641-1970]
10-19	38 132 (20.2)	1826 (20.6)	24 846 (19.3) [23 188-26 503]	1175 (19.5) [1052-1297]
20-49	29 850 (15.8)	1422 (16)	28 252 (22) [26 341-30 163]	1322 (22) [1190-1454]
50-99	13 461 (7.1)	640 (7.2)	14 129 (11) [12 832-15 427]	660 (11) [567-753]
100-499	13 975 (7.4)	664 (7.5)	14 979 (11.6) [13 932-16 026]	699 (11.6) [612-787]
≥500	6805 (3.6)	324 (3.6)	7525 (5.8) [6813-8238]	352 (5.9) [292-412]
Primary treatment				
AS/WW	58 575 (31)	2770 (31.2)	33 106 (25.7) [31 469-34 742]	1560 (25.9) [1430-1690]
AA	8776 (4.6)	417 (4.7)	8367 (6.5) [7798-8936]	385 (6.4) [326-445]
GnRH	52 789 (27.9)	2513 (28.3)	53 127 (41.3) [50 891-55 363]	2484 (41.3) [2311-2658]
RP	41 134 (21.8)	1856 (20.9)	16 695 (13) [15 194-18 196]	757 (12.6) [658-856]
RT	27 610 (14.6)	1327 (14.9)	17 407 (13.5) [15 679-19 135]	828 (13.8) [719-937]

Abbreviations: AA, antiandrogen treatment; AS/WW, active surveillance or watchful waiting; GnRH, gonadotropin-releasing hormone agonist/antagonist; PSA, prostate-specific antigen; RP, radical prostatectomy; RT, radiotherapy.

### Prostate Cancer–Specific Mortality

**Figure 3.**
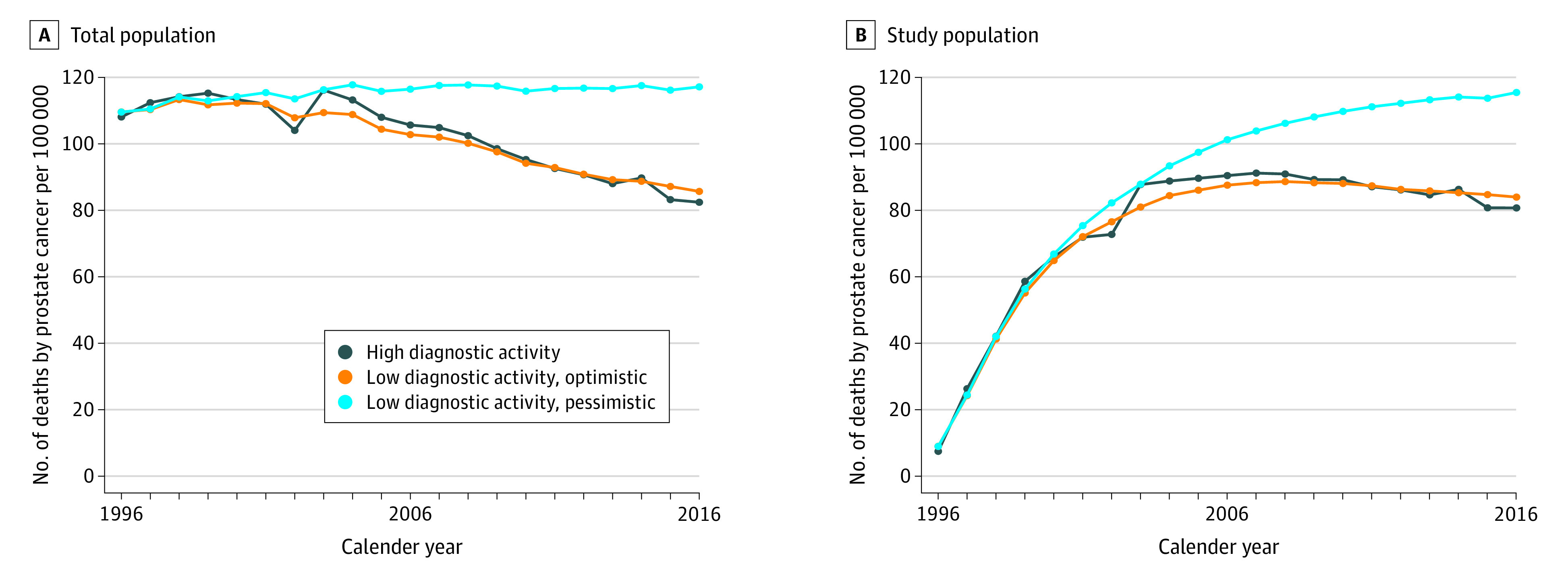
Prostate Cancer–Specific Death The Proxy-Based Risk-Stratified Incidence Simulation Model–Prostate Cancer model was used to calculate prostate cancer mortality. The graphs show results per 100 000 men, age-standardized (age distribution of men 40 to 100 years in 2016). Prevalent cases with diagnosis before 1996 excluded in panel B.

When comparing prostate cancer mortality between the 2 scenarios, results ranged from no difference (IRR, 1.02; 95% CI, 0.98-1.05) in the optimistic extreme to 13 less deaths per 100 000 men per year (IRR, 0.85; 95% CI, 0.82-0.88) in the pessimistic extreme, depending on which of the 2 extreme assumptions on lead time and treatment effects was used (Figure 3). This represents up to 15% fewer deaths in the scenario with high diagnostic activity.

## Discussion

In this simulation cohort study, high diagnostic activity was associated with an increase in the incidence of low- and intermediate-risk prostate cancer, as well as a substantial increase in the number of men receiving deferred and curative treatment. In contrast, the decreases in incidence of advanced or metastasized disease and in prostate cancer mortality were modest.

A high diagnostic activity was associated with a substantial increase in the number of men who were diagnosed with prostate cancer; however, this difference was mainly seen in low- and intermediate-risk disease. Considering the low risk of prostate cancer–specific death,^10,11^ this likely represents a considerable overdiagnosis. Furthermore, because the diagnostic accuracy of transrectal ultrasonography-guided prostate biopsies is poor,^12^ the number of men who underwent biopsies is much higher. These results are consistent with the findings of a systematic review by Fenton et al,^13^ which concluded that an increased diagnostic activity (the introduction of PSA screening) reduced prostate cancer mortality but was associated with overdiagnosis and possibly overtreatment.^1^

There were large differences in the number of men who received curative treatment between the 2 scenarios, with more than twice as many men in the scenario with high diagnostic activity. This increase likely has a large impact on the health-related quality of life for these men as negative side effects from treatments such as erectile dysfunction, urinary leakage, or bowel problems are common.^14,15^ In the LAPPRO-trial, a contemporary study on functional outcomes after prostate cancer treatment, 19% of men who had undergone robotically assisted radical prostatectomy by experienced surgeons suffered from incontinence and 68% suffered from erectile dysfunction.^15^

Regarding the occurrence of advanced or metastasized disease, only small differences were seen between the two scenarios, with slightly less men in the scenario with a high diagnostic activity. It is possible that our timeframe is too short to detect a larger difference in the incidence of advanced or metastasized disease. However, these results are in line with the large, randomized clinical trials on prostate cancer screening that showed slightly fewer men with advanced disease among screened men.^16^ Although fewer men with advanced or distant metastasized disease were seen in the scenario with high diagnostic activity, almost no difference was seen in the number of men receiving hormonal treatment.

We compared the prostate cancer–specific mortality between the 2 scenarios over these 20 years and found a modest difference, with fewer deaths in the scenario with high diagnostic activity. These differences are likely to increase with longer follow-up as illustrated in Figure 3. Still, we consider the differences in prostate cancer–specific death to be modest in comparison with the large differences seen in the number of diagnosed and treated men. Although not directly comparable, these results are in line with a review and meta-analysis of several of the large randomized controlled trials on prostate cancer screening that found no or only modest benefits in terms of prostate cancer–specific mortality.^13,16^ However, it is important to point out that one of the included trials, the ERSPC trial found a significant, 20% decrease in prostate cancer mortality at 16-year follow-up among screened men.^17^

### Limitations and Strengths

The limitations of this study include the use of a simulation model. Our study therefore only provides an overview and not exact data for the scenario with low diagnostic activity. Changes in exposure to unknown etiological factors could affect the underlying risk, and these factors cannot be captured by our model approach. Furthermore, the benefits of a higher diagnostic activity in terms of reduced prostate cancer mortality increases over time (as seen in Figure 3), so the benefits of high diagnostic activity would probably be greater in a future evaluation.

It is important to point out that we did not evaluate the effects of PSA testing per se but rather the increased overall diagnostic activity driven by the introduction of PSA, such as transrectal ultrasound-guided biopsies and higher number of biopsy cores taken per session. Neither did we simulate or evaluate an organized screening program.

The strengths of this study include the nationwide, population-based design, the data with almost complete coverage, the long follow-up time, and the use of a new simulation model, PRISM-PC.^7^ There are preexisting simulation models, such as the Microsimulation Screening Analysis (MISCAN) model^18^ or the Fred Hutch Cancer Research Center (FHCRC) model.^19^ These simulation models are based on assumptions on the natural history of the cancer, test properties, and effects of repeated testing. Moreover, model parameters are often calibrated using a variety of selected data material. In contrast to previous simulation models for prostate cancer, our model uses historical and observed data for prostate cancer and simulates real-life scenarios rather than clinical trials.

## Conclusions

The opportunistic PSA-driven diagnostic activity that has occurred over the past 30 years was associated with a large increase in the number of men diagnosed with prostate cancer. Most of them had low- and intermediate-risk disease with long life expectancy even without treatment. The high diagnostic activity was associated with a 2-fold increase in curative treatment and a modest decrease in mortality. In the future, new diagnostic methods should be introduced to clinical practice in an organized manner to assess the effect of these methods.

## Notes

Supplement. **eAppendix.** Detailed Description of the Simulation Model PRISM-PC
