# Supplementary material for: Variation in Prostate-Specific Antigen Testing Rates and Prostate Cancer Treatments and Outcomes in a National 20-Year Cohort
Source: JAMA Netw Open. 2021 May 17;4(5):e219444. doi: 10.1001/jamanetworkopen.2021.9444 (PMC8129820; doi:10.1001/jamanetworkopen.2021.9444)
Supplement: Supplement. — eAppendix. Detailed Description of the Simulation Model PRISM-PC [file jamanetwopen-e219444-s001.pdf]

Supplemental Online Content

Bergengren O, Westerberg M, Holmberg L, Stattin P, Bill-Axelson A, Garmo H. Variation in prostate-specific antigen testing rates and prostate cancer treatments and outcomes in a national 20-year cohort. *JAMA Netw Open.* 2021;4(5):e219444. doi:10.1001/jamanetworkopen.2021.9444

eAppendix. Detailed Description of the Simulation Model PRISM-PC

This supplemental material has been provided by the authors to give readers additional information about their work.

© 2021 Bergengren O et al. *JAMA Network Open.*

eAppendix. Detailed Description of the Simulation Model PRISM-PC

Simulating incidence and prevalence of prostate cancer

All men aged 40 to 100 living in Sweden during the period 1992 to 2016 were included to estimate model parameters, while men living in Sweden from 1996-2016 were included in the analysis. Number of men of specific age each calendar year were retrieved from Statistics Sweden (http://www.statistikdatabasen.scb.se). The men were divided in two groups *No prostate cancer* and *Prostate cancer*. The latter group was identified using data from Prostate Cancer data Base Sweden (PCBaSe version 4.0) [1].

At start of simulation the *No prostate cancer* group consist of all men in Sweden not diagnosed with prostate cancer aged 40 to 100 and the *Prostate cancer* group consist of all prevalent prostate cancer cases in Sweden 31/12 1995. The simulation runs in one-year time steps. In each time step new men aged 40 are entering the group *No prostate cancer*. For each man the simulation determines

a) the men in the *No prostate cancer* group that will be diagnosed with prostate cancer during the year, and those that die without a prostate cancer diagnosis during the year,

b) a prostate cancer risk category R1=Low risk, R2=Intermediate risk, R3=High risk, R4=regionally metastatic, R5=distant metastases for those diagnosed with prostate cancer during the year,

c) the men in the *Prostate cancer* group that will die during the year,

d) the cause of death for men in the *Prostate cancer* group that die during the year.

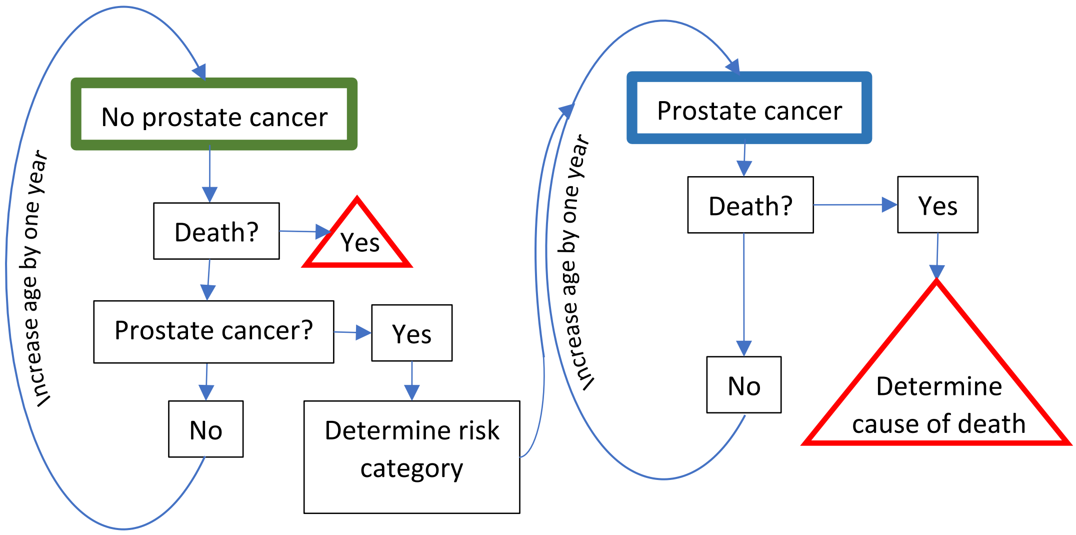

Figure A1. Graphical illustration of the state transition model. The red triangles indicate absorbing states, the green rectangle indicate the pool of men alive and free from prostate cancer, and the dark blue rectangle indicate the pool of men alive and living with a prior prostate cancer diagnosis.

© 2021 Bergengren O et al. *JAMA Network Open.*

Model for outcome “Death?” amongst *No prostate cancer* men

The model for risk of death amongst men in the group *No prostate cancer* is based on data from men free of prostate cancer (n=842 536) randomly selected from the background population in PCBaSe4.0 and depends on age and calendar period. For each such comparison man we collected data on date of inclusion in PCBaSe, year of birth, last day of follow up (LDOF), follow up time and a censoring variable (1= death, 0=censored).

Table A1. Follow up data for 20 men, out of a total of 842 536 comparison men, in PCBaSe4.0.

Inclusion date	Year of birth	Quarter of birth	LDOF	CENSOR	TimeFU
2010-03-31	1940	1	2016-12-31	0	6.7545
2004-09-06	1938	1	2005-08-05	0	0.9117
2016-02-10	1950	4	2016-12-31	0	0.8898
2007-03-07	1933	2	2007-11-09	1	0.6763
2016-12-13	1947	3	2016-12-31	0	0.0493
2002-04-16	1921	2	2002-12-15	1	0.6653
2013-02-16	1946	2	2016-12-31	0	3.8714
2005-10-26	1919	1	2016-12-31	0	11.181
2012-03-14	1941	4	2016-12-31	0	4.7996
2009-03-25	1945	2	2016-12-31	0	7.7702
2001-07-04	1914	3	2004-07-16	1	3.0336
2014-11-20	1959	2	2016-12-31	0	2.1137
2007-11-22	1931	2	2016-12-31	0	9.1091
1999-11-19	1923	2	1999-12-17	1	0.0767
2008-08-15	1932	1	2016-12-31	0	8.3781
2016-03-08	1957	4	2016-12-31	0	0.8159
2003-10-22	1926	2	2016-12-31	0	13.194
2006-11-07	1945	3	2016-12-31	0	10.149
2011-05-10	1936	2	2016-12-31	0	5.6456
2012-03-07	1939	4	2016-12-31	0	4.8187
⋮	⋮	⋮	⋮	⋮	⋮

The data in Table A1 was transformed into long format by splitting on calendar year (variable name *currentyear*) and age (variable name *currentage*). On the data transformed to long format we applied the model presented in Table A2.

Table A2. R-model describing the probability of death during one year for the *No prostate cancer* men.

```
library(mgcv)

noPCa_outflow_model <- bam(CENSOR ~ currentyear + te(currentage,k=10,
                    family=binomial, data = noPCa_outflow_data)
```

© 2021 Bergengren O et al. *JAMA Network Open.*

Model for "Prostate cancer?" amongst *No prostate cancer* men

The model of the risk of being diagnosed with prostate cancer and the models for classification of risk category R1, R2, R3, R4, R5 (low risk, intermediate risk, high risk, regionally metastatic, distant metastases) are governed by a predetermined level of diagnostic activity, age, and history of diagnostic activity. Data from 236 506 men diagnosed between 1970 and 2016 gathered in PCBaSe4.0 is used as input to these models. A sample of diagnosis data is presented in Table A3.

Table A3. Example of risk category data from 15 men out of 236 506 diagnosed between 1970 and 2016 used to determine prostate cancer status and risk categories R1, R2, R3, R4 and R5.

id	age	DX-date	censor	time	County	Risk category
1	66	2004-02-13	0=Censor	11,406	Stockholm	R3
2	56	2005-12-19	0=Censor	10,188	Stockholm	R2
3	69	2012-01-13	0=Censor	3,225	Skåne	R1
4	89	1999-05-08	1=PC death	6,065	Jämtland	R5
5	58	2014-05-19	0=Censor	1,793	Jönköping	R2
6	72	1997-03-13	2=Other death	10,295	Stockholm	R1
7	74	2004-07-02	2=Other death	7,929	Skåne	**NA**
8	60	2003-10-10	0=Censor	12,047	Stockholm	R1
9	69	2000-11-30	2=Other death	3,801	Stockholm	R3
10	57	2013-11-15	0=Censor	2,653	Stockholm	R2
11	61	2013-01-04	0=Censor	2,314	Stockholm	R1
12	60	2000-09-09	2=Other death	4,487	Stockholm	**NA**
13	54	1997-12-13	1=PC death	8,624	Västra Götaland	R2
14	72	2015-01-15	0=Censor	0,145	Uppsala	R4
15	80	2000-12-20	2=Other death	3,340	Stockholm	**NA**
⋮	⋮	⋮	⋮	⋮	⋮	⋮

Missing *risk category* was imputed using multiple imputing by chained equations and m=20 imputation datasets were created [2]. Next, we combined each imputation dataset with data on number of men living in each of the 21 counties of Sweden. Parts of this population size data is displayed in Table A4 and can be retrieved in open access from Statistics Sweden http://www.statistikdatabasen.scb.se/pxweb/en/ssd/START__BE__BE0101__BE0101A/FolkmangdNov/ .

By combining each imputation dataset created from data in Table A3 with data as displayed in Table A4 we create a data set including the proxy for diagnostic activity 0-20 years back in time denoted M0, M1, …, M20, for each combination of county (N=21), age (40, 41,…,100), calendar year (1992, 1997, …, 2016). Number of events and men at risk are aggregated as displayed in Table A5. Number of men diagnosed with prostate cancer in risk categories 1-5 are denoted R1, …, R5. The risk of being diagnosed with prostate cancer is calculated using the models presented in Table A6

© 2021 Bergengren O et al. *JAMA Network Open.*

Table A4. Total number of men of specific age by year and county. Data available from Statistics Sweden

		Year														
**County**	**Age**	…...	**2002**	**2003**	**2004**	**2005**	**2006**	**2007**	**2008**	**2009**	**2010**	**2011**	**2012**	**2013**	**2014**	**2015**	**2016**
Stockholm	40	…...	14280	14962	16342	16130	16092	15787	14962	14905	15514	16400	16898	16880	17206	16533	16413
Stockholm	41	…...	13802	14182	14858	16266	16174	16178	15807	15055	14963	15648	16506	16960	16957	17246	16657
Stockholm	42	…...	13150	13645	14082	14812	16238	16168	16193	15848	15072	15103	15738	16543	17042	16943	17331
Stockholm	43	…...	12996	13078	13573	14056	14835	16244	16187	16236	15845	15131	15151	15799	16633	17031	17007
Stockholm	44	…...	12847	12958	13010	13538	14040	14821	16252	16189	16166	15945	15224	15181	15899	16701	17152
Stockholm	45	…...	12876	12771	12890	12975	13523	14022	14838	16260	16172	16192	15960	15292	15229	15906	16778
Stockholm	46	…...	12840	12816	12726	12850	12969	13529	14020	14897	16268	16214	16248	15990	15354	15202	15953
Stockholm	47	…...	12173	12802	12735	12662	12816	12973	13484	14046	14878	16312	16272	16288	16055	15383	15222
Stockholm	48	…...	11863	12108	12711	12674	12631	12825	12975	13533	14029	14942	16325	16314	16330	16021	15426
Stockholm	49	…...	11885	11801	12070	12653	12664	12661	12789	13002	13535	14076	14963	16304	16308	16301	16064
⋮	⋮	⋮	⋮	⋮	⋮	⋮	⋮	⋮	⋮	⋮	⋮	⋮	⋮	⋮	⋮	⋮	⋮
Stockholm	90	…...	838	804	872	877	914	1015	978	1071	1275	1312	1183	1285	1278	1351	1268
Stockholm	91	…...	642	690	656	697	707	738	845	803	872	1038	1064	979	1051	1070	1112
Stockholm	92	…...	441	493	536	531	554	543	574	692	649	705	847	872	805	846	873
Stockholm	93	…...	329	342	386	408	416	410	417	439	526	513	539	674	675	634	680
Stockholm	94	…...	217	254	255	291	315	310	303	311	333	384	381	419	512	515	484
Stockholm	95	…...	163	156	183	175	217	222	213	221	219	259	277	270	333	379	381
Stockholm	96	…...	100	109	106	130	130	151	157	146	154	145	185	199	188	244	275
Stockholm	97	…...	70	65	83	70	91	96	97	107	100	115	90	125	139	132	162
Stockholm	98	…...	33	48	50	57	56	56	68	65	72	72	79	64	81	95	81
Stockholm	99	…...	29	22	30	34	37	31	39	44	46	41	48	63	39	51	62
Stockholm	100+	…...	32	39	33	47	53	53	51	54	60	66	61	63	71	70	63

© 2021 Bergengren O et al. *JAMA Network Open.*

⋮	⋮	⋮	⋮	⋮	⋮	⋮	⋮	⋮	⋮	⋮	⋮	⋮	⋮	⋮	⋮	⋮	⋮

Table A5. Example of data used for estimation of incidence models for risk categories R1, R2, R3, R4 and R5.

**birth year**	**county**	**age**	**year**	**n_at_risk***	**R1**	**R2**	**R3**	**R4**	**R5**	**M0#**	**M1#**	**M2#**	**M3#**	**M4#**	**M5#**	**M6#**	**M7#**	**M8#**	**M9#**	**M10#**	**M11#**	**M12#**	**M13#**	**M14#**	**M15#**	**M16#**	**M17#**	**M18#**	**M19#**	**M20#**
⋮	⋮	⋮	⋮	⋮	⋮	⋮	⋮	⋮	⋮	⋮	⋮	⋮	⋮	⋮	⋮	⋮	⋮	⋮	⋮	⋮	⋮	⋮	⋮	⋮	⋮	⋮	⋮	⋮	⋮	⋮
1940	Blekinge	56	1996	893	0	0	0	0	0	2.2	1.4	0.8	0.6	0.5	0.3	0.3	0.2	0.2	0.1	0.1	0.0	0.0	0.0	0.0	0.0	0.0	0.4	0.4	0.4	0.4
1940	Blekinge	57	1997	891	0	1	0	0	0	3.0	2.2	1.4	0.8	0.6	0.5	0.3	0.3	0.2	0.2	0.1	0.1	0.0	0.0	0.0	0.0	0.0	0.0	0.4	0.4	0.4
1940	Blekinge	58	1998	885	0	0	0	0	0	4.2	3.0	2.2	1.4	0.8	0.6	0.5	0.3	0.3	0.2	0.2	0.1	0.1	0.0	0.0	0.0	0.0	0.0	0.0	0.4	0.4
1940	Blekinge	59	1999	884	0	0	0	0	1	7.0	4.2	3.0	2.2	1.4	0.8	0.6	0.5	0.3	0.3	0.2	0.2	0.1	0.1	0.0	0.0	0.0	0.0	0.0	0.0	0.4
1940	Blekinge	60	2000	885	0	0	0	0	0	11.9	7.0	4.2	3.0	2.2	1.4	0.8	0.6	0.5	0.3	0.3	0.2	0.2	0.1	0.1	0.0	0.0	0.0	0.0	0.0	0.0
1940	Blekinge	61	2001	881	2	1	1	1	0	17.5	11.9	7.0	4.2	3.0	2.2	1.4	0.8	0.6	0.5	0.3	0.3	0.2	0.2	0.1	0.1	0.0	0.0	0.0	0.0	0.0
1940	Blekinge	62	2002	874	0	2	0	0	0	24.3	17.5	11.9	7.0	4.2	3.0	2.2	1.4	0.8	0.6	0.5	0.3	0.3	0.2	0.2	0.1	0.1	0.0	0.0	0.0	0.0
1940	Blekinge	63	2003	877	2	0	1	1	0	32.0	24.3	17.5	11.9	7.0	4.2	3.0	2.2	1.4	0.8	0.6	0.5	0.3	0.3	0.2	0.2	0.1	0.1	0.0	0.0	0.0
1940	Blekinge	64	2004	867	1	2	0	0	0	38.7	32.0	24.3	17.5	11.9	7.0	4.2	3.0	2.2	1.4	0.8	0.6	0.5	0.3	0.3	0.2	0.2	0.1	0.1	0.0	0.0
1940	Blekinge	65	2005	851	0	1	2	1	0	47.5	38.7	32.0	24.3	17.5	11.9	7.0	4.2	3.0	2.2	1.4	0.8	0.6	0.5	0.3	0.3	0.2	0.2	0.1	0.1	0.0
1940	Blekinge	66	2006	840	2	2	0	0	1	57.8	47.5	38.7	32.0	24.3	17.5	11.9	7.0	4.2	3.0	2.2	1.4	0.8	0.6	0.5	0.3	0.3	0.2	0.2	0.1	0.1
1940	Blekinge	67	2007	823	2	4	0	1	1	60.3	57.8	47.5	38.7	32.0	24.3	17.5	11.9	7.0	4.2	3.0	2.2	1.4	0.8	0.6	0.5	0.3	0.3	0.2	0.2	0.1
1940	Blekinge	68	2008	802	2	1	1	2	0	56.9	60.3	57.8	47.5	38.7	32.0	24.3	17.5	11.9	7.0	4.2	3.0	2.2	1.4	0.8	0.6	0.5	0.3	0.3	0.2	0.2
1940	Blekinge	69	2009	782	3	2	1	2	0	54.1	56.9	60.3	57.8	47.5	38.7	32.0	24.3	17.5	11.9	7.0	4.2	3.0	2.2	1.4	0.8	0.6	0.5	0.3	0.3	0.2
1940	Blekinge	70	2010	764	2	3	2	0	2	52.9	54.1	56.9	60.3	57.8	47.5	38.7	32.0	24.3	17.5	11.9	7.0	4.2	3.0	2.2	1.4	0.8	0.6	0.5	0.3	0.3
1940	Blekinge	71	2011	743	2	4	3	2	0	53.8	52.9	54.1	56.9	60.3	57.8	47.5	38.7	32.0	24.3	17.5	11.9	7.0	4.2	3.0	2.2	1.4	0.8	0.6	0.5	0.3

© 2021 Bergengren O et al. *JAMA Network Open.*

1940	Blekinge	72	2012	722	1	3	2	0	1	56.0	53.8	52.9	54.1	56.9	60.3	57.8	47.5	38.7	32.0	24.3	17.5	11.9	7.0	4.2	3.0	2.2	1.4	0.8	0.6	0.5
1940	Blekinge	73	2013	697	3	3	4	1	0	57.0	56.0	53.8	52.9	54.1	56.9	60.3	57.8	47.5	38.7	32.0	24.3	17.5	11.9	7.0	4.2	3.0	2.2	1.4	0.8	0.6
1940	Blekinge	74	2014	684	2	0	1	0	2	57.3	57.0	56.0	53.8	52.9	54.1	56.9	60.3	57.8	47.5	38.7	32.0	24.3	17.5	11.9	7.0	4.2	3.0	2.2	1.4	0.8
⋮	⋮	⋮	⋮	⋮	⋮	⋮	⋮	⋮	⋮	⋮	⋮	⋮	⋮	⋮	⋮	⋮	⋮	⋮	⋮	⋮	⋮	⋮	⋮	⋮	⋮	⋮	⋮	⋮	⋮	⋮

*not including incident, censored, or those that died, during the corresponding year. #columns have been multiplied with 10 000 and rounded to one decimal place and corresponds to the proxy M0-M20 for diagnostic activity 0-20 years back in time.

© 2021 Bergengren O et al. *JAMA Network Open.*

Table A6. R-code for calculation of probability of being diagnosed with prostate cancer during one year for the *No prostate cancer* men. DATA corresponds to the data set in Table A5.

Setup data sets

```
# create time lag matrix
X <- matrix(seq(0,-20,length=21),nrow(DATA),21,byrow=TRUE)
# create age matrix
A <- c()
for(i in 1:21){A <- cbind(A,DATA$age)}
X <- X + A # create lagged age matrix
L  <- as.matrix(DATA[,paste0(type,"_",0:20)]) # Proxy (now and past)
Lb <- as.matrix(DATA[,paste0("M",1:20)])  # Proxy (past)
Xb <- X[,2:21] # Age (past)
Xn <- X[,1] # Age (now)
Ln <- as.matrix(DATA[,paste0("M",0)]) # Proxy (now)
Lb <- log(Lb) # log proxy (past)
Ln <- log(Ln) # log proxy (now)
```

Knots for splines

```
knotlist=list( "Xb"=c(35,45,55,65,70,75,80,85,90,95),
               "Xn"=c(45,55,65,70,75,80,85,90,95),
               "Ln"=c(-15,-12,-11,-10,-8,-7,-6,-4),
               "Lb"=c(-15,-12,-11,-10,-8,-7,-6,-4))
```

Incidence models

```
require(mgcv)
```

Risk category 1

```
incidence_model_1 <- gam( cbind(DATA[,paste0("R",1)],DATA$n_at_risk ) ~
                      te(Xn,Ln,k=c(length(knotlist$Xn),length(knotlist$Ln)) ,bs="cs") +
                      te(Xb,Lb,k=c(length(knotlist$Xb), length(knotlist$Lb)),bs="cs"),
                      family=binomial, knots=knotlist, method="REML", select=TRUE)
```

Risk category 2

```
incidence_model_2 <- gam( cbind(DATA[,paste0("R",2)],DATA$n_at_risk ) ~
                      te(Xn,Ln,k=c(length(knotlist$Xn),length(knotlist$Ln)) ,bs="cs") +
                      te(Xb,Lb,k=c(length(knotlist$Xb), length(knotlist$Lb)),bs="cs"),
                      family=binomial, knots=knotlist, method="REML", select=TRUE)
```

Risk category 3

```
incidence_model_3 <- gam( cbind(DATA[,paste0("R",3)],DATA$n_at_risk ) ~
                      te(Xn,Ln,k=c(length(knotlist$Xn),length(knotlist$Ln)) ,bs="cs") +
                      te(Xb,Lb,k=c(length(knotlist$Xb), length(knotlist$Lb)),bs="cs"),
                      family=binomial, knots=knotlist, method="REML", select=TRUE)
```

Risk category 4

```
incidence_model_4 <- gam( cbind(DATA[,paste0("R",4)],DATA$n_at_risk ) ~
                      te(Xn,Ln,k=c(length(knotlist$Xn),length(knotlist$Ln)) ,bs="cs") +
                      te(Xb,Lb,k=c(length(knotlist$Xb), length(knotlist$Lb)),bs="cs") ,
                      family=binomial, knots=knotlist, method="REML", select=TRUE)
```

Risk category 5

```
incidence_model_5 <- gam( cbind(DATA[,paste0("R",5)],DATA$n_at_risk ) ~
                      te(Xn,Ln,k=c(length(knotlist$Xn), length(knotlist$Ln)) ,bs="cs") +
                      te(Xb,Lb,k=c(length(knotlist$Xb),length(knotlist$Lb)),bs="cs") ,
                      family=binomial, knots=knotlist, method="REML", select=TRUE)
```

Model for “Death?” amongst *Prostate cancer* men

Using the imputed data created from data in Table A1, splitting on calendar year and age and put in long format the models presented in Table A7 are applied. The estimation is performed stratified by risk category R1, …., R5.

© 2021 Bergengren O et al. *JAMA Network Open.*

Table A7. R-code for calculation of probability of death from prostate cancer and other during one year for the *Prostate cancer* men. DATA corresponds to the data set in Table A3 transformed to long format.

Knots for splines

```
knotslist = list(timefu     = c(0,1,5,10,15),
                 age        = c(50,60,70,80,90),
                 year       = c(1992,1996,2004,2012))
```

Other cause of death

```
Retf2 <- DATA[DATA$censor %in% c(0,2),]
Retf2$censor[Retf2$censor %in% 2]=1

Mod2<- bam(censor ~ te(age,year,timefu,    k=c(length(knotslist$age),
length(knotslist$year),length(knotslist$timefu))    ,bs="cs"),
              data= Retf2,
              knots=knotslist,
              family=binomial(),
              discrete=TRUE,
              select=TRUE
     )
```

Prostate cancer death

```
Retf1 <- DATA[DATA$censor %in% c(0,1),]

Mod1 <- bam(censor ~ te(age,year,timefu,    k=c(length(knotslist$age),
length(knotslist$year),length(knotslist$timefu))    ,bs="cs"),
              data= Retf1,
              knots=knotslist,
              family=binomial(),
              discrete=TRUE,
              select=TRUE
     )
```

Simulating treatment trajectories

From the simulation we collect age at diagnosis, year of diagnosis, risk category, survival time, and cause of death (or indication of censoring) for each man diagnosed with prostate cancer as displayed in Figure A2. To determine the treatment trajectory, we create a dataset with all observed treatment trajectories in PCBaSe4.0. To create an observed treatment trajectory for each simulated man we select men with similar characteristics and randomly select an observed treatment trajectory. The selection is performed with replacement using weighted probabilities. A higher probability was given to men in the observed that were more like the simulated fictive case.

© 2021 Bergengren O et al. *JAMA Network Open.*

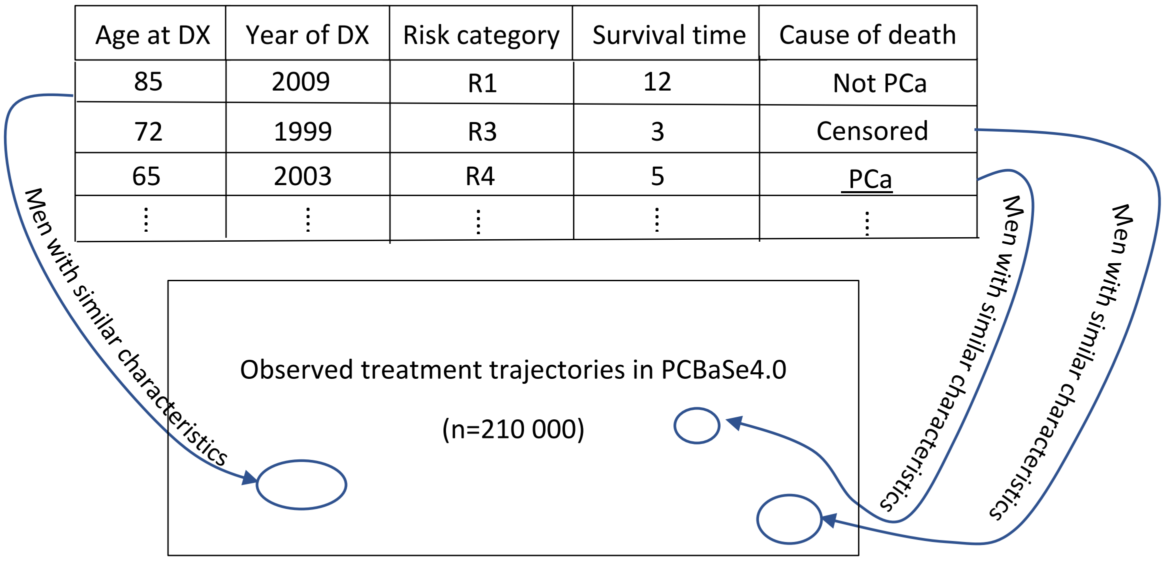

Figure A2. Illustration of the resampling method used to determine trajectories for the pool of men alive with a prior prostate cancer diagnosis.

Observed treatments for prostate cancer were divided into deferred treatment (DT), radical prostatectomy (RP), radio therapy (RT), and androgen deprivation therapy (ADT). Data on treatment trajectories were obtained in PCBaSe^{Traject} [3] for each man diagnosed with prostate cancer between 1996-2016. Treatment changes were retrieved from the National Prostate Cancer Registry (NPCR), RetroRad [4], Prescribed Drug Registry, and the Patient Registry.

A limitation in PCBaSe^{Traject} is the possibility to detect transitions to ADT prior to 2006 when the Prescribed Drug Registry was introduced. Therefore, ADT treatment started prior to 2006 had an unknown start date unless it's the primary treatment. We imputed missing data using multiple imputation [2,5].

1. Van Hemelrijck M, Wigertz A, Sandin F, et al. Cohort Profile: the National Prostate Cancer Register of Sweden and Prostate Cancer data Base Sweden 2.0. *International journal of epidemiology.* 2013;42(4):956-967.
2. Buuren Sv. *Flexible imputation of missing data.* Boca Raton, FL: CRC Press; 2012.
3. Van Hemelrijck M, Garmo H, Wigertz A, Nilsson P, Stattin P. Cohort Profile Update: The National Prostate Cancer Register of Sweden and Prostate Cancer data Base--a refined prostate cancer trajectory. *International journal of epidemiology.* 2016;45(1):73-82.
4. Beckmann K, Garmo H, Nilsson P, Franck Lissbrant I, Widmark A, Stattin P. Radical radiotherapy for prostate cancer: patterns of care in Sweden 1998-2016. *Acta Oncol.* 2020;59(5):549-557.
5. Rubin DB. *Multiple imputation for nonresponse in surveys.* Hoboken, N.J: John Wiley; 2011.

© 2021 Bergengren O et al. *JAMA Network Open.*
